# Study protocol for a randomized controlled trial: tongue strengthening exercises in head and neck cancer patients, does exercise load matter?

**DOI:** 10.1186/s13063-015-0889-5

**Published:** 2015-09-04

**Authors:** Gwen Van Nuffelen, Leen Van den Steen, Olivier Vanderveken, Pol Specenier, Carl Van Laer, Diane Van Rompaey, Cindy Guns, Steven Mariën, Marc Peeters, Paul Van de Heyning, Jan Vanderwegen, Marc De Bodt

**Affiliations:** Department of Otolaryngology and Head & Neck Surgery – Rehabilitation Center for Communication Disorders, Antwerp University Hospital, Antwerp, Belgium; Faculty of Medicine and Health Sciences, University of Antwerp, Antwerp, Belgium; Department Medical Oncology, Antwerp University Hospital, Antwerp, Belgium; University College Thomas More, Antwerp, Belgium; Department of Otolaryngology and Head & Neck Surgery, UMC Sint-Pieter, Brussels, Belgium; Faculty of Speech, Pathology and Audiology, Ghent University, Ghent, Belgium

**Keywords:** dysphagia, pressure, rehabilitation, resistance, strength, tongue, training

## Abstract

**Background:**

Reduced tongue strength is an important factor contributing to early and late dysphagia in head and neck cancer patients previously treated with chemoradiotherapy. The evidence is growing that tongue strengthening exercises can improve tongue strength and swallowing function in both healthy and dysphagic subjects. However, little is known about the impact of specific features of an exercise protocol for tongue strength on the actual outcome (strength or swallowing function). Previous research originating in the fields of sports medicine and physical rehabilitation shows that the degree of exercise load is an influential factor for increasing muscle strength in the limb skeletal muscles. Since the tongue is considered a muscular hydrostat, it remains to be proven whether the same concepts will apply.

**Methods/Design:**

This ongoing randomized controlled trial in chemoradiotherapy-treated patients with head and neck cancer investigates the effect of three tongue strengthening exercise protocols, with different degrees of exercise load, on tongue strength and swallowing. At enrollment, 51 patients whose dysphagia is primarily related to reduced tongue strength are randomly assigned to a training schedule of 60, 80, or 100 % of their maximal tongue strength. Patients are treated three times a week for 8 weeks, executing 120 repetitions of the assigned exercise once per training day. Exercise load is progressively adjusted every 2 weeks. Patients are evaluated before, during and after treatment by means of tongue strength measurements, fiber-optic endoscopic evaluation of swallowing and quality-of-life questionnaires.

**Discussion:**

This randomized controlled trial is the first to systematically investigate the effect of different exercise loads in tongue strengthening exercise protocols. The results will allow the development of more efficacious protocols.

**Trial registration:**

Current Controlled Trials ISRCTN14447678.

## Background

Chronic oropharyngeal dysphagia is a common sequela in head and neck cancer survivors after chemoradiotherapy [1–5]. Several studies have found that up to 70 % of this population still have dysphagia 12 months after the end of treatment [3–10]. Prolonged dysphagia in patients with head and neck cancer is commonly the result of a downward spiral in which muscle disuse and a never-ending cascade of chemoradiotherapy-induced tissue fibrosis are the main etiological factors [4, 5]. Subsequent loss of muscle function and strength results in chronic, often late-onset, swallowing problems that have both life-threatening potential and a major negative impact on quality of life [1, 3–10]. Several studies and publications identify reduced tongue strength as an important mechanism of dysphagia in this population [1, 11, 12]. Tongue strength is the main bolus driving force, and reduced tongue strength is related to pharyngeal residue and aspiration [1, 13–17]. Tongue strengthening exercises can improve tongue strength and swallowing function in both healthy and pathological populations, including patients with chronic dysphagia following chemoradiotherapy [18–25]. Although the number of studies documenting positive outcomes has increased gradually, the total number of subjects included remains limited, especially when considering the subgroup of patients. Other studies, moreover, do not support these results fully [24, 26, 27]. It can be hypothesized that the outcome of tongue strengthening exercises depends on a number of influencing factors, such as the composition and execution of the exercise protocol. Indeed, several studies within the fields of sports medicine and physical rehabilitation show that the effect of exercises on skeletal muscle strength depends on exercise protocol features, such as the number of repetitions, frequency of practice, and exercise load [28–31]. These findings led to the formulation of ‘principles of exercise’ or ‘principles of strength training,’ which represent the guidelines for efficient exercise protocols [28, 32, 33]. However, evidence in support of a straightforward application of these principles to the oropharyngeal muscles – and more specifically, to the tongue– is lacking at present [34, 35]. The tongue is a unique muscle structure, a muscle hydrostat (that is, a muscular organ composed almost entirely of muscle and lacking typical systems of skeletal support) with differences in muscle fiber composition and function, compared with other skeletal muscles [34, 36–38] that might induce different responses to strengthening exercises.

So far, studies comparing different protocols for tongue strengthening exercises are sparse and generally performed in healthy populations. A study by Clark *et al.* [20] in 39 healthy subjects revealed no differences in outcome between a concurrent (three types of exercise in each therapy session) and sequential lingual strength training protocol (a 3 week sequence of the different types of exercise). In addition, the principle of training specificity was refuted (meaning that elevation, protrusion, or lateralization exercises had equal and mutual effects). The same authors, however, indicated some degree of specificity regarding tongue strength, endurance, power, and speed in a subsequent study group of 25 healthy adults [19]. Regarding training tools, no difference in outcome was demonstrated using either a tongue depressor or the Iowa Oral Performance Instrument in healthy adults [19]. Considering training form, an ongoing study by Steele and colleagues [39] is investigating the hypothesis that tongue strengthening exercises could yield better outcomes for swallowing liquids in stroke patients if the exercises were modified to focus on tasks with similar pressure profiles to those seen in liquid swallowing in healthy people, taking into account both strength and timing (that is, skills training). Unfortunately, none of these studies methodically investigated the effect of different degrees of exercise load or resistance (often expressed as a percentage of the 1-repetition maximal force capacity (1-RM)). It is generally accepted that exercises targeting skeletal muscles that do not force the neuromuscular system beyond the level of usual activity will not elicit adaptations, and consequently, the system must be overloaded progressively (that is, the resistance should increase stepwise), to make continual gains [28, 34]. The appropriate degree of resistance in skeletal muscle training depends on the goal of treatment. Strength training is often performed at high resistance levels (80–100 % 1-RM), whereas improving muscle power requires lower levels of resistance (0–60 % 1-RM) [19, 28, 30, 31]. A meta-analysis by Rhea *et al.* [40] demonstrated 80 % 1-RM to be most effective for improving muscle strength in trained individuals, whereas 60 % 1-RM elicits maximal gains in untrained individuals [34, 40]. Thus, it can be assumed that gains in strength are greater or achieved faster when practicing at higher resistance levels. However, some studies suggest that training at more than 60 % 1-RM might cause overuse injuries, especially in the inactive population or when resting periods are not respected [34, 40].

It should be noted that tongue strength improvement was documented in several studies, each using different levels of resistance. The therapy protocol used by Robbins *et al.* [21, 22] comprises multiple repetitions at 60 % 1-RM during the first week and 80 % 1-RM during the following 7 weeks, including a progressive overload by a biweekly determination of 1-RM. Other studies found improved tongue strength following tongue strengthening exercises performed at maximal effort without reporting overuse injuries [18, 20, 25]. Even exercise schedules with randomly chosen resistance levels for each repetition that varied between 20 and 90 % of the patient’s maximum isometric capacity, were found to improve tongue strength [23, 24]. Since these studies not only address different populations but also vary in a number of methodological aspects, it is not possible to draw any definitive conclusions regarding preferential exercise load for tongue strengthening exercises. The goal of the proposed randomized controlled trial (RCT) is to systematically investigate the effect of levels of resistance on tongue strength and the subsequent swallowing function in dysphagic patients with head and neck cancer previously treated with chemoradiotherapy.

## Methods/Design

### Study objectives

The goal of this study is to investigate the differences in tongue strength gain between three tongue strengthening exercise protocols that only differ by the levels of resistance used with every repetition (60, 80, or 100 % of the patient’s 1-RM).

### Study design

**Fig. 1:**
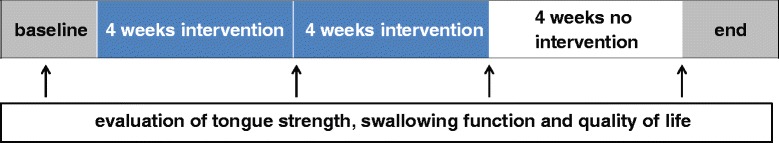
Outline of the trial

The outline of this RCT is presented in Fig. 1. Subjects are evaluated prior to treatment, after 4 and 8 weeks of treatment, and 4 weeks after the last therapy session. The maximal interval between baseline evaluation and the start of therapy is 1 week. Evaluations during and after therapy will tolerate a margin of 48 hours at the most to accommodate transport and office schedules. All measurements, the twice weekly determinations of 1-RM, and supervision of the treatment will be carried out by experienced speech language pathologists. Subjects perform three therapy sessions per week (totaling 24 sessions) on nonconsecutive days to allow for sufficient rest periods. Each therapy session consists of 120 tongue presses: 60 anterior repetitions and 60 posterior repetitions. These 60 repetitions are divided into 12 sets of five repetitions with obligatory rest periods of 60 s between sets. Therapy sessions start in alternating order with either anterior or posterior repetitions. Subjects are randomly assigned to group 1 (levels of resistance at 100 % 1-RM), group 2 (levels of resistance at 80 % 1-RM), or group 3 (levels of resistance at 60 % 1-RM). In accordance with the principle of progressive overload, 1-RM is determined at baseline and subsequently every 2 weeks during training.

**Fig. 2:**
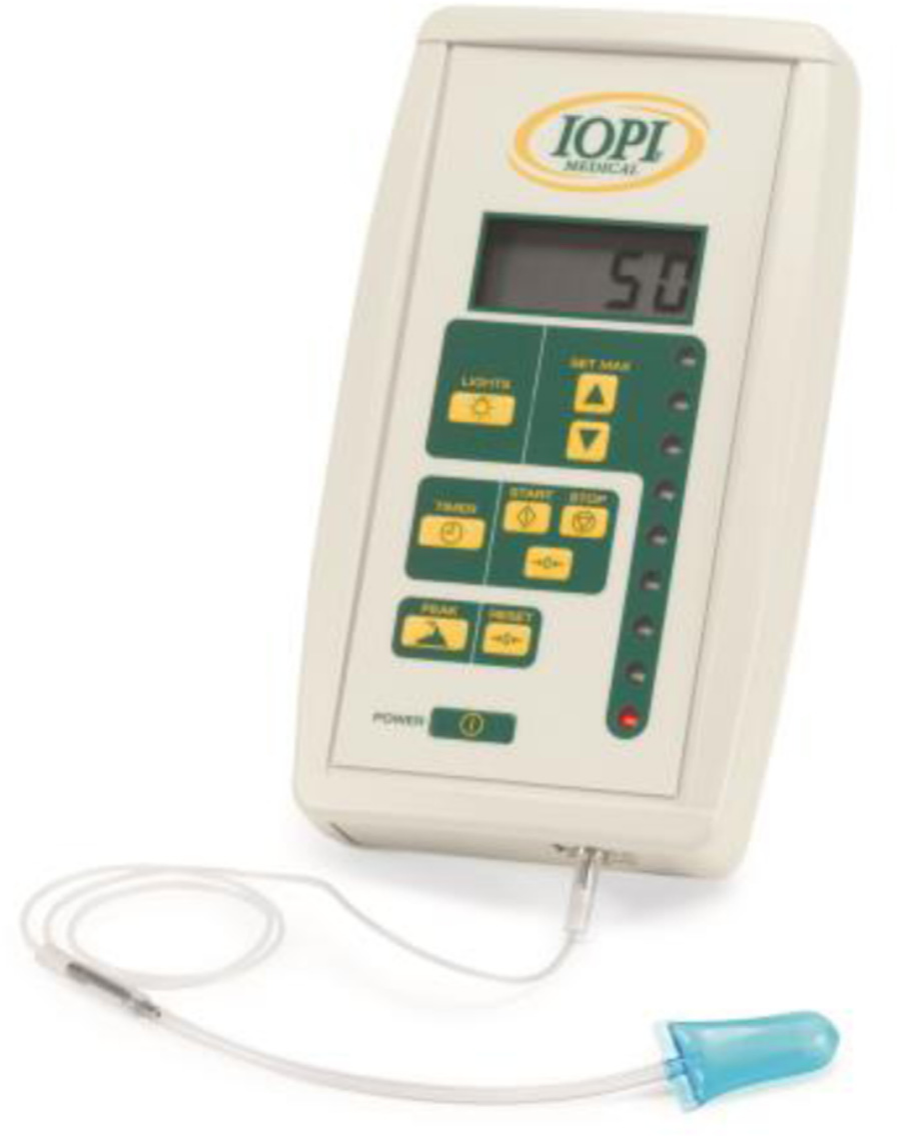
Iowa Oral Performance Instrument (image duplicated with permission of Tara Hart, CEO of IOPI Medical LLC)

Exercises are performed using the Iowa Oral Performance Instrument (model 2.2, Fig. 2). This is a handheld manometer attached to an air-filled bulb. A digital display readout shows the amount of pressure generated by squeezing the bulb with the tongue, expressed in kilopascals (kPa). The device allows the user to set a target value or target levels of resistance manually (for example, 80 % 1-RM). Visual feedback of the pressure exerted is provided by a vertical series of light emitting diodes in which the uppermost light corresponds to 100 % of the target levels of resistance. Subjects are instructed to push the tongue bulb against the palate as hard as necessary according to the selected resistance and hold this amount of effort for 3 s. During the anterior repetitions, the proximal end of the bulb is positioned immediately behind the upper teeth at the midline of the palate. Posterior positioning is done by placing the distal tip of the bulb at the transition between hard and soft palate, again at the midline of the palate. A permanent mark on the connecting tube just anterior to the incisors assures accurate placement for each repetition and measurement.

### Study population

This study is carried out in chemoradiotherapy-treated patients with head and neck cancer who have chronic dysphagia (that is, dysphagia that has been present for at least 1 month and no earlier than 6 months after the last day of radiation treatment) that is at least partly attributable to insufficient tongue strength. Insufficient tongue strength is defined as the presence of residue at the base of tongue or in the valleculae, as diagnosed during fiber-optic endoscopic evaluation of swallowing (FEES) at baseline. A person’s age- and sex-specific maximum isometric tongue pressure (MIP) is not a crucial factor for inclusion; this means that subjects with MIP values within the 95 % prediction interval of established normative data [41] are not excluded. Candidates for enrollment are both men and women older than 18 years without cognitive, language, motor, hearing, or visual deficits that could interfere with the correct execution of the training. Exclusion criteria are a history of major oral or head and neck surgery and neurological disorders with an impact on oral function or swallowing (including stroke, traumatic brain injury, Parkinson’s disease, amyotrophic lateral sclerosis). Concurrent oral motor exercises or swallowing maneuvers to improve swallowing are not allowed during the study period.

### Randomization

Subjects are assigned to one of the three therapy groups based on a sequence generated by the online randomization tool at www.randomizer.org. Clinicians involved in the inclusion procedure are blinded to this assignment by using numbered and sealed envelopes.

### Limitations of the study

The inclusion of a control group would obviously strengthen the proposed study design. The authors decided to leave out this additional study arm, owing to concerns with feasibility and given the exploratory character of this RCT. Further support for this decision is the result of a previous study showing a significantly higher effect on MIP in experimental groups performing tongue strengthening exercises than in a control group, even with a relatively small sample size [42].

Another possible limitation of this RCT is the lack of stratification. Overall, stratification was not considered because of the exploratory character of this RCT. With regards to the most evident stratification factors, ‘radiation dose’ and ‘tumor characteristics’ [43–45], it should be noted that inclusion is based on functional swallowing deficits, implicating underlying RCT-induced neuromuscular failure in all subjects regardless of tumor characteristics or radiation dose.

### Outcome measures

The outcome measures in this RCT are divided into three main categories: (1) tongue strength measurements, (2) evaluation of the swallowing function, and (3) evaluation of quality of life. A team of experienced speech language pathologists and otolaryngologists will perform the evaluations.

#### Tongue strength measurements

To obtain MIP (expressed as kPa), the participant pushes the tongue bulb of the Iowa Oral Performance Instrument as hard as possible against the palate for 3 s, both at the anterior and posterior positions (as described in ‘Study design’). The highest value of three trials is considered the MIP and will be used for further analysis. The MIP is the primary outcome variable of interest.

#### Swallowing function

Swallowing function is evaluated using a comprehensive FEES examination, the Mann Assessment of Swallowing Ability – Cancer (MASA-C) [46], the Functional Oral Intake Scale [47] and a self-evaluation. For the latter, a 100 mm visual analogue scale is used, with the ends defined as ‘I can’t swallow’ (0) and ‘I don’t have any swallowing difficulties’ (100). Both the FEES and MASA-C are conducted with four different bolus types: 5 and 10 ml of thin liquid, and 5 and 10 ml of yogurt. Each bolus type is administered three times. Outcome measures for FEES are the Penetration-Aspiration-Scale [48], the Carnaby Videofluoroscopy Evaluation scales for dysphagia and aspiration [49], the Pooling-score [50] and the Boston Residue and Clearance Scale (BRACS) [51]. The latter will be completed for each bolus type after the first and third bolus. Patients with a score of 1 or higher for the BRACS items ‘base of tongue’ or ‘valleculae’ at baseline, as judged by an experienced clinician, can be considered for inclusion. All FEES examinations will be digitally recorded for data analysis. Separate video clips per participant and per bolus type will be randomized and judged by two blinded and experienced clinicians.

#### Quality of life

Swallowing-related quality of life will be surveyed by means of the Dutch version of the Swallowing Quality-of-Life Questionnaire [52] and the Dysphagia Handicap Index [53].

### Sample size calculation

The sample size calculation is based on the primary outcome variable (that is, MIP) and performed using GLIMMPSE [54]. With evolution of the mean values based on preliminary data of previous studies investigating the effect of tongue strengthening exercises on the evolution of MIP (SD MIPa 4.2 kPa; SD MIPp 4.5 kPa ; basic correlation 0.4 and decay rate 0.5) [25, 45] a total sample size of 45 participants (15 per group), not taking into account dropouts, is needed to demonstrate a statistically significant effect of levels of resistance at a *P* value of 0.05 and a power of 0.8 when using repeated measures with Geisser–Greenhouse correction (3 groups × 4 time points). The targeted total sample size is 51.

### Data analysis

Data will be analyzed using a repeated measures analysis with post-hoc testing, using the most recent version of IBM SPSS Statistics (V22.0) and R software.

### Study sites

This is a single-center study. The study is carried out at the Rehabilitation Center for Communication Disorders of the Antwerp University Hospital, Belgium.

### Ethical approval

This research protocol was reviewed and approved by the Ethical Committee of the Antwerp University Hospital and the University of Antwerp (Ethisch Comité van het Universitair Ziekenhuis Antwerpen en de Universiteit Antwerpen, 14/24/253). The Belgian registration number is B300201421549. Informed consent is obtained from each participant.

## Discussion

There is growing evidence that tongue strengthening exercises can improve tongue strength and swallowing function in both healthy and dysphagic subjects, including patients with head and neck cancer treated with chemoradiotherapy. There is still a need for additional insight in how to develop the most efficient tongue strengthening exercise protocol. The planned RCT will provide supplemental information on which to base clinical decisions during swallowing rehabilitation in people with head and neck cancer. These concepts could also be useful when treating patients who do not have head and neck cancer but who have dysphagia related to reduced tongue strength.

## Trial status

Enrollment is currently ongoing. The target completion date is 2016.

## Abbreviations

1-RM: 1-repetition maximal force capacity
BRACS: Boston Residue and Clearance Scale
FEES: fiber-optic endoscopic evaluation of swallowing
MASA-C: Mann Assessment of Swallowing Ability – Cancer
MIP: maximum isometric tongue pressure
RCT: randomized controlled trial
